# Application of Tourniquet Does Not Influence Early Clinical Outcomes After Total Knee Arthroplasty

**DOI:** 10.7759/cureus.12435

**Published:** 2021-01-02

**Authors:** Ejaz A Chaudhry, Amer Aziz, Ahmad Faraz, Mohammad Iqbal, Muhammad Yasir Tarar, Syed Hasan Mustafa Rizvi, Noah Khan, Muhammad Tahir, Chadi Ali

**Affiliations:** 1 Trauma and Orthopaedics, Royal Berkshire NHS Trust, Reading, GBR; 2 Orthopaedics, Ghurkhi Hospital Trust, Lahore, PAK; 3 Spine Surgery, Ghurki Trust Teaching Hospital, Lahore, PAK; 4 Trauma and Orthopaedics, Leeds Teaching Hospitals NHS Trust, Leeds, GBR; 5 Orthopaedics and Trauma, Royal Shrewsbury and Telford Hospital NHS Trust, Shrewsbury, GBR; 6 Trauma and Orthopaedics, Services Institute of Medical Sciences, Lahore, PAK; 7 Trauma and Orthopaedics, Blackpool Teaching Hospitals, NHS Foundation Trust, Blackpool, GBR; 8 Internal Medicine, Peterborough City Hospital, Peterborough, GBR; 9 Trauma and Orthopaedics, Royal Victoria Hospital, Belfast, GBR; 10 Orthopaedics, Jinnah Postgraduate Medical Centre, Karachi, PAK; 11 Spine Surgery, Royal National Orthopaedic Hospital, London, GBR

**Keywords:** tourniquet, total knee replacement, pain

## Abstract

Introduction

The use of a tourniquet during total knee arthroplasty (TKA) is still a topic of debate, given the conflicting results in the literature with respect to complications, pain, functional outcome, and blood loss. However, due to a lack of convincing data on early patient-reported outcomes (PROMS), this study aims to compare pain and functional outcomes in total knee arthroplasty patients with and without a tourniquet.

Methods

A randomized controlled trial was set up at a tertiary care hospital that spanned from 1^st^ February 2015 to 31^st^ July 2018. We included all primary total knee arthroplasties performed for patients aged between 50 and 80 years. Oxford Knee Score (OKS), Numerical Pain Rating Score (NPRS), Visual Analogue Scale (VAS) for satisfaction, active range of knee motion, and Short Form-12 Survey (SF-12) scores were collected pre-surgery and then at six-weeks and six-months interval with a p-value of 0.05 considered to be significant.

Results

Two hundred and forty patients participated in the study; 117 patients were randomized to surgery with the tourniquet inflated and 123 to surgery with the tourniquet deflated. There were 43.4% males, and 56.6% females in the tourniquet inflated group with an average age of 62.29±9.63 years while in tourniquet deflated group, there were 46.7% males and 53.3% females with a mean age of 65.41±9.042 years (p-value for age is 0.404; the p-value for gender is 0.086).

Despite the increase in intraoperative blood loss in both the groups, there was no significant increase in blood transfusions as both groups recorded the need for postoperative blood transfusion - 12 patients in the tourniquet group and 19 in the non-tourniquet group, but this difference was statistically insignificant (p=0.231). The perioperative blood loss was significantly lower (p<0.001) in the tourniquet group (490.29±47.752) compared to in the non-tourniquet group (526.18±12.796), while the duration of surgery was comparable in both groups (p=0.156).

The length of stay for the two groups did not statistically differ (p=0.976) - the mean length of stay for the tourniquet group was 6.16±2.38 days and for the non-tourniquet group it was 6.18±2.34 days.

There were no significant differences between the two groups regarding patient-reported outcomes (PROMS) at six-weeks and six-months. However, during the in-hospital stay, only the NPRS score for knee pain showed that the non-tourniquet patients had a lower NPRS compared to the tourniquet group and this difference was statistically significant (p=0.02). During the postoperative hospital stay, there was no significant difference among the two treatment groups for VAS, OKS, SF-12, and range of motion (flexion/extension).

At the six weeks follow-up, both groups had similar outcomes for the range of movements and pain scores. Besides, no difference was noted among the tourniquet and non-tourniquet groups even after a follow-up of six months. Regarding complications, 27 patients in the tourniquet group did complain of numbness during the study period compared to 10 in the non-tourniquet group (p=0.001).

Conclusion

In conclusion, a tourniquet application helps minimize intraoperative blood loss and results in a faster procedure. Furthermore, the application of the tourniquet is safe and effective and does not affect the functional outcomes and pain scale in total knee arthroplasty.

## Introduction

Total knee arthroplasty (TKA) is a highly effective surgical procedure for managing moderate-to-severe degenerative joint disease [1]. However, surgeons aim to optimize clinical results and to reduce complications associated with the operation. The use of a tourniquet during TKA is still a topic of debate, given the conflicting results in the literature concerning complications, pain, functional outcome, and blood loss [1-3]. Therefore, the use of a tourniquet for TKA is widely variable among arthroplasty surgeons [4, 5]. Thus, the objective of this randomized trial is to compare the clinical outcomes of TKA between the tourniquet group and non-tourniquet group of patients and to see if there is a difference during the early phase of rehabilitation after TKA.

## Materials and methods

**Figure 1:**
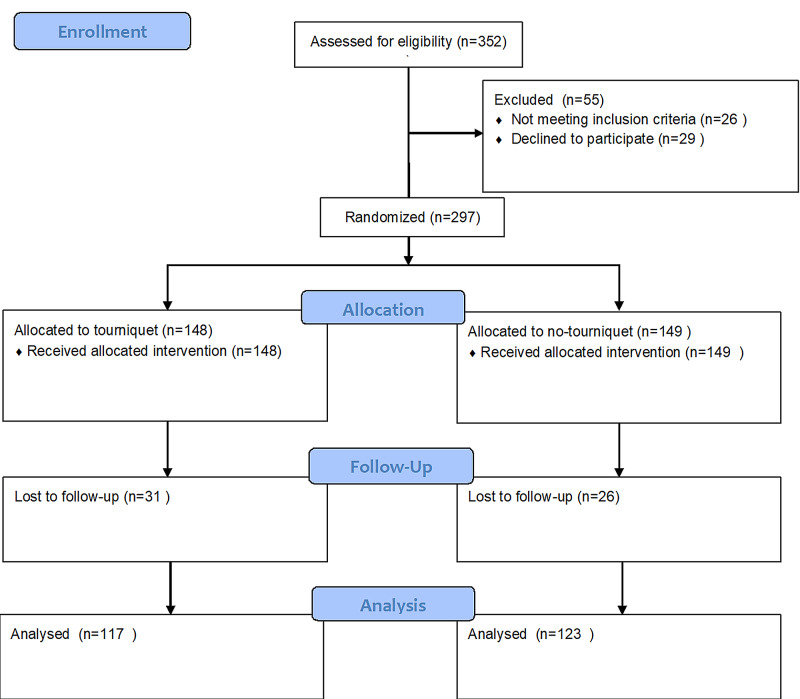
Flow diagram of the study

After the approval from the institutional review board committee, a randomized controlled trial was set up at a tertiary care charity hospital that spanned from 1st February 2015 to 31st July 2018. The study was registered in the Chinese Clinical Trial Registry under the trial number ChiCTR2000033173 and conducted in accordance with the Helsinki declaration with an informed patient consent with the outcomes being reported according to the Consolidated Standards of Reporting Trials (CONSORT) guidelines (Figure 1) [6].

Inclusion and exclusion criteria

We included all primary total knee arthroplasties performed for grade 3 and 4 osteoarthritides according to Kellgren and Lawrence System [7, 8], aged between 50 and 80 years having a physical status American Society of Anesthetist (ASA) score I, II, III [9].

We excluded patients previously operated for any open knee surgery assessed on history, revision arthroplasty, body mass index greater than 40 kg/m^2^, vascular calcifications or a documented diagnosis of peripheral vascular disease, patients with a bleeding disorder, functionally limiting spine disease, other functionally limiting lower extremity disease, patients who cannot perform baseline functional assessments, any allergy or contraindication to study protocol medications.

Data collection and randomization

Patients’ demographics and baseline clinical data such as Numerical Pain Rating Score (NPRS) [10] for knee pain, Visual Analogue Scale (VAS) for satisfaction [11], Oxford Knee Scale (OKS) [12], knee flexion extension range, and quality of life assessment Short Form-12 (SF-12) [13, 14], were prospectively collected one week prior to the surgery at the time of preoperative consultation and anesthesia fitness. A higher OKS, VAS, and SF-12 indicated greater levels of function, while a lower number in NPRS denoted less pain.

The participants were undergoing 1:1 computer-generated random allocation stratified by age, gender, and body mass index, into either tourniquet or non-tourniquet group. All operations were performed under epidural anesthesia. The patients received intravenous cefuroxime (1.5 g) 30-minutes before induction of anesthesia and twice postoperatively. In addition, a single dose of 1 gram of intravenous tranexamic acid was administered prior to the incision. The envelope for the cohort assignment was opened just before the incision. An independent team of assessors was allocated to collect the data, and they were also blinded to the group allocation.

Interventions

Those operating within the tourniquet group had a tourniquet pressure between 250 to 300 mmHg depending on the operating surgeon’s preference. Tourniquet was not continuously inflated for longer than 90 minutes in the lower limb to minimize skin necrosis risk. Tourniquet was deflated just before closure to identify and to coagulate major bleeding points.

Patients in the no tourniquet group had an injection of the 2% lidocaine and epinephrine according to the safe dose of 7 mg/ml/kg prior to the incision for vasoconstriction of the blood vessels in the subcutaneous tissues.

The mean amount of blood loss was calculated post-operatively using the number of sponges soaked and blood volume in the suction bottle.

All the patients followed the same postoperative protocol. They had local anesthesia in the wound and in the subcutaneous tissue prior to wound closure as per weight. The postoperative pain protocol included an alternative regimen of intravenous paracetamol and ibuprofen every six hours. While during the discharge period, oral paracetamol and celecoxib were prescribed for pain. For in-hospital venous thromboembolism prophylaxis, both mechanical and chemoprophylaxis were prescribed. At the time of discharge, all patients were instructed to take 81 mg of oral aspirin twice a day for thirty days unless they were taking another anticoagulant preoperatively.

During the in-hospital stay, the nurses were instructed to record the NPRS for knee pain at six to eight hours increment, and the physiotherapist was asked to document the functional assessments and range of motion during the patient's hospital stay.

Outcomes

The clinical outcomes were collected at six weeks and at six months. The clinical outcomes recorded were OKS, NPRS, range of motion of the knee, VAS for satisfaction, and SF-12 quality health indicator.

Data analysis

Data analysis was done using SPSS version 23 (IBM Inc., Armonk, USA). The analysis between the two groups for continuous variables was performed via independent t-test and Mann-Whitney U-test, and the p-value of less than 0.05 was considered statistically significant. For qualitative variables such as complications, Chi-square and Fischer exact tests were used, and a p-value of 0.05 was considered as significant.

## Results

A total number of two hundred and forty patients participated in the study; 117 patients were randomized to surgery with the tourniquet inflated and 123 to surgery with the tourniquet deflated. There were 43.4% males, and 56.6% were females in the tourniquet inflated group with an average age of 62.29±9.63 years, while in the tourniquet deflated group, there were 46.7% males and 53.3% females with a mean age of 65.41±9.042 years (p-value for age is 0.404 and for gender is 0.086). The BMI for the tourniquet group was 30.18±0.69 kg/m^2^, whereas, for the non-tourniquet group, the BMI was 30.81±2.09kg/m^2^ (p=0.103). Both the groups were evenly matched in their patient demographics.

The perioperative blood loss was significantly lower (p<0.001) in the tourniquet group, while the duration of surgery was comparable in both groups (p=0.156), as indicated in Table 1.

**Table 1:** Intraoperative parameters P-value is calculated using Mann-Whitney U-test.

Intraoperative measurements	Tourniquet	No tourniquet	p-value
Blood loss (milliliters)	490.29±47.752	526.18±12.796	0.001
Operating time (minutes)	110.00±10.225	113.24±8.246	0.156

The length of stay for the two groups did not statistically differ (p=0.976) as the mean length of stay for the tourniquet group was 6.16±2.38 days and for the non-tourniquet group was 6.18±2.34 days.

There were no significant differences between the two groups in terms of patient-reported outcomes (PROMS) at six-weeks and six-months, as shown in Table 2. However, during the in-hospital stay, only the NPRS score for knee pain showed a significant difference (p=0.02).

**Table 2:** Patient-reported outcomes Independent t-test and Mann-Whitney U-test (*) were used to calculate the p-values. NPRS - Numerical Pain Rating Score; OKS - Oxford Knee Score; VAS - Visual Analogue Scale; SF-12 - Short Form-12

Variable	Tourniquet	No tourniquet	p-value
Baseline			
NPRS	6.23±0.75	5.95±1.10	0.228
VAS satisfaction	16.03±1.834	16.12±1.665	0.836
OKS	29.76 ±9.56	29.50±8.74	0.522
Knee flexion extension range	82.09±3.671	81.97±3.572	0.894
SF-12	34.09±4.100	33.53±4.769	0.606
In-hospital			
NPRS	3.44±0.77	2.90±0.92	0.02
VAS satisfaction	32.08±2.99	32.32±3.39	0.763
OKS	33.41±3.88	32.44±3.99	0.313
Knee flexion extension range	90.05±10.51	88.85±8.44	0.83
Six weeks			
NPRS	3.91±0.26	3.83±0.36	0.342
VAS satisfaction	50.50±6.91	49.61±5.71	0.568
OKS	35.06±3.33	35.18±3.77	0.892
Knee flexion extension range	95.32±6.82	99.38±7.29	0.021
SF-12	34.29±4.28	35.12±3.54	0.391
Six months			
NPRS	2.66±0.18	2.60±0.21	0.21
VAS satisfaction	73.05±11.84	70.23±16.85	0.721*
OKS	39.02±1.21	39.14±1.20	0.691
Knee flexion extension range	105.88±3.608	106.74±3.203	0.306
SF-12	39.32±1.471	39.35±2.102	0.947

During the postoperative hospital stay, there was no significant difference between the two treatment groups in VAS, OKS, range of motion (flexion/extension), and SF-12. At the six weeks follow-up, both groups had similar outcomes regarding the range of movements and pain scores. In addition, no difference was noted among the tourniquet and non-tourniquet groups even after a follow-up of six months, as shown in Table 2.

Regarding complications, we did not record any events of venous thromboembolism or any complication that resulted in an unplanned admission or return to theatre. However, six patients in the tourniquet group and eight in the non-tourniquet had minor surgical site infections during the in-hospital period that were treated successfully with antibiotics (p=0.649). In addition, 27 patients in the tourniquet group complained of numbness during the study period as compared to 10 in the non-tourniquet group (p=0.001).

Despite an increase in intraoperative blood loss, there was no significant increase in blood transfusions as both groups recorded the need for postoperative blood transfusion - 12 patients in the tourniquet group and 19 in the non-tourniquet group, but this difference was statistically insignificant (p=0.231). 

## Discussion

The present study demonstrates that there was no statistically significant difference for pain and PROMS among tourniquet and non-tourniquet groups. However, those managed with tourniquet had significantly lower blood loss than non-tourniquet ones.

Although various randomized clinical trials have been published, the use of a tourniquet during total knee joint replacement has still been a topic of debate [15-17]. There is conflicting evidence regarding the effect of tourniquet use on perioperative blood loss and postoperative function and pain. Dennis et al. reported that patients who underwent concomitant bilateral TKA with a tourniquet for a single knee and not on the contralateral side endured muscle weakness postoperatively on the side where the tourniquet was used after three months of surgery [18]. However, we did not inspect the muscle mass and contractile forces; we did analyze the functional outcomes of operatively treated limb, suggesting no significant difference among the two treatment groups.

Huang et al. found that knee pain was significantly higher in inpatient until postoperative day five. They also observed that range of motion at discharge among patients without a tourniquet had a greater range of motion than those where TKA was done with the use of a tourniquet [19]. Chen et al. observed a similar trend, with patients who underwent TKA with a tourniquet reporting notably higher knee pain until the third postoperative day [20]. Our study shows there was a significant difference between the two groups in NPRS during the hospital stay, but no remarkable difference was noted at six weeks or six months follow-up. Furthermore, VAS satisfaction and OKS did not show any consequential difference between the two groups during the hospital stay or at follow-up.

Smith and Hing did not find tourniquet useful in TKA as it did not decrease the requirement of blood transfusion [21]. A meta-analysis on 11 randomized trials conducted by Rama et al. concluded that early release of the tourniquet was associated with greater perioperative blood loss in comparison to when the tourniquet was released after the closure of the wound [22]. Schnettler et al. discovered a paradoxical relationship between tranexamic acid and tourniquet; his study demonstrated an increase in blood loss using a tourniquet and tranexamic acid, compared when tranexamic acid was used alone [23].

However, Goel et al. observed a decrease in the perioperative blood loss after the use of a tourniquet, which was clinically relevant [17]. Likewise, our trial supported the findings of Goel et al., and we reported a statistically significant reduction in perioperative blood loss (p=0.001, Table 1) in the tourniquet group, which was clinically relevant, as 19 patients required blood transfusions during the postoperative period in the non-tourniquet group as opposed to 12 in the tourniquet group, however, this was statistically insignificant (p=0.231).

Mori et al. reported that the use of tourniquet was associated with a higher risk of deep venous thrombosis (DVT) after TKA [24]; however, we did not report any incident of venous thromboembolism in our study.

The use of a tourniquet can lead to long-term postoperative complications. A 2016 cohort study that observed surgical complications proposed that an increased tourniquet time was associated with an increased risk of 30-day readmission rate [25]. In addition, Tie et al. observed a higher risk of wound infections among patients who had extended tourniquet times [26]. In our study, the operating time was marginally higher in the non-tourniquet group than those who had the operation with the tourniquet, but this difference was not significant (p=0.156). Our study did not show statistically significant wound complications (p=0.649) in the current study as only six patients in the tourniquet group and eight in the non-tourniquet had minor wound complications during the in-hospital period and were treated successfully with antibiotics.

There were some limitations to this randomized control trial. Firstly, this study was not multicentered because of limited resources. Also, we could have included some more variables such as the Timed Up and Go (TUG) test, the stair climb test, and comparing the girth of the quadriceps compared with the unoperated side to add more variables and that would have contributed to a robust study. Despite the following shortcomings, our study did have the following strengths. Firstly, it was a randomized trial. Secondly, the use of validated patient-reported outcomes strengthens the study and allows for future investigators to compare their results with ours.

## Conclusions

In conclusion, the application of a tourniquet helps minimize intraoperative blood loss and results in a faster procedure. The application of the tourniquet is safe and effective and does not affect the functional outcomes in total knee arthroplasty in the early rehabilitation period.
